# RETRACTION: A Systematic Review of Nursing Students' Attitude and Related Factors Towards Pressure Ulcer Prevention

**DOI:** 10.1111/iwj.70255

**Published:** 2025-02-25

## Abstract

**RETRACTION:** HermisA. H. , MollaeiA. , VajargahP. G. , KarkhahS. , TakasiP. , FiroozM. , HosseiniS. J. , OtaghvarH. A. , and RazianiY. , “A Systematic Review of Nursing Students' Attitude and Related Factors Towards Pressure Ulcer Prevention,” International Wound Journal 20, no. 8 (2023): 3404–3416, 10.1111/iwj.14191.37434034 PMC10502266

The above article, published online on 11 July 2023, in Wiley Online Library (http://onlinelibrary.wiley.com/), has been retracted by agreement between the journal Editor in Chief, Professor Keith Harding; and John Wiley & Sons Ltd. Following an investigation by the publisher, all parties have concluded that this article was accepted solely on the basis of a compromised peer review process. The editors have therefore decided to retract the article. The authors disagree with the retraction.

**RETRACTION:** A. H. Hermis , A. Mollaei , P. G. Vajargah , S. Karkhah , P. Takasi , M. Firooz , S. J. Hosseini , H. A. Otaghvar , and Y. Raziani , “A Systematic Review of Nursing Students' Attitude and Related Factors Towards Pressure Ulcer Prevention,” International Wound Journal 20, no. 8 (2023): 3404–3416, 10.1111/iwj.14191.37434034 PMC10502266

The above article, published online on 11 July 2023, in Wiley Online Library (http://onlinelibrary.wiley.com/), has been retracted by agreement between the journal Editor in Chief, Professor Keith Harding; and John Wiley & Sons Ltd. Following an investigation by the publisher, all parties have concluded that this article was accepted solely on the basis of a compromised peer review process. The editors have therefore decided to retract the article. The authors disagree with the retraction.

